# Formation Mechanism of Residual Stresses in Micro-Injection Molding of PMMA: A Molecular Dynamics Simulation

**DOI:** 10.3390/polym12061368

**Published:** 2020-06-17

**Authors:** Can Weng, Tao Ding, Mingyong Zhou, Jiezhen Liu, Hao Wang

**Affiliations:** 1 College of Mechanical and Electrical Engineering, Central South University, Changsha 410083, China; dingtao@csu.edu.cn (T.D.); zmy_csu@163.com (M.Z.); jishe@csu.edu.cn (J.L.); 2 State Key Laboratory of High Performance Complex Manufacturing, Central South University, Changsha 410083, China; 3 Department of Mechanical Engineering, Faculty of Engineering, National University of Singapore, 9 Engineering Drive 1, Singapore 117575, Singapore; mpewhao@nus.edu.sg

**Keywords:** injection molding, residual stress, PMMA, molecular dynamics simulation, aspect ratio

## Abstract

Injection molding is an economical and effective method for manufacturing polymer parts with nanostructures and residual stress in the parts is an important factor affecting the quality of molding. In this paper, taking the injection molding of polymethyl methacrylate (PMMA) polymer in a nano-cavity with an aspect ratio of 2.0 as an example, the formation mechanism of residual stresses in the injection molding process was studied, using a molecular dynamics simulation. The changes in dynamic stress in the process were compared and analyzed, and the morphological and structural evolution of molecular chains in the process of flow were observed and explained. The effects of different aspect ratios of nano-cavities on the stress distribution and deformation in the nanostructures were studied. The potential energy, radius of gyration and elastic recovery percentage of the polymer was calculated. The results showed that the essence of stress formation was that the molecular chains compressed and entangled under the flow pressure and the restriction of the cavity wall. In addition, the orientation of molecular chains changed from isotropic to anisotropic, resulting in the stress concentration. At the same time, with the increase in aspect ratio, the overall stress and deformation of the nanostructures after demolding also increased.

## 1. Introduction

Functional surfaces with micro/nano-structures are widely used in microfluidic chips [1,2], superhydrophobic surfaces [3], optical conversion surfaces [4] and biomimetic surfaces [5] due to their excellent optical, electrochemical and biological properties. Therefore, micro/nano manufacturing technology has become the forefront of modern science and technology. At present, micro/nano size and high precision requirements pose great challenges to processing and manufacturing. Micro-injection molding is an ideal means for manufacturing various shapes and sizes of micro/nano structured parts, which is economic, effective and has the potential for mass production [6]. However, residual stress is one of the most important factors that influences the final quality of the injection molded parts. Its existence will directly affect the mechanical, optical and service properties of the parts, and it may even lead to cracks in the parts [7,8]. Therefore, many scholars have been eager to resolve the residual stresses of surface micro/nano structured parts via injection molding.

As we all know, the ordered orientation of molecules indicates the existence of residual stresses [9,10], which can be reflected by birefringence experiments [11,12]. So far, in the process of injection molding, the influences of processing parameters on residual stresses have been well analyzed through experiments and finite element simulations, among which temperature and pressure are the research points of most concern. Weng et al. [13] used the birefringence method to detect residual stresses in polycarbonate (PC) micro-lens arrays and found that the mold temperature was the most significant factor affecting residual stress. Generally, the melt could fill the mold cavity smoothly without forming a frozen layer with an appropriate mold temperature [14]. Lin et al. [15] studied the effects of the processing conditions on the birefringence characteristics of Fresnel lenses and stated that the residual stress was sensitive to the melt temperature. Sara et al. [16] proposed that the packing pressure also had a significant effect on residual stresses. Besides this, residual stresses could be effectively reduced by an annealing treatment [17].

The influence of processing parameters on residual stress and the molding quality of micro-injection molding is different from that of traditional injection molding. When the scale is reduced to micro/nano-size, the scale effect and specific surface area are increased. Therefore, some macroscopic mechanisms are difficult to explain using microscopic laws and phenomena [18]. In this way, molecular dynamics simulation has the advantage of analyzing macroscopic mechanisms at the molecular level. At present, molecular dynamics simulation is widely used in the study of flow behavior [19,20,21], interfacial heat transfer [22,23,24] and interfacial adhesion [25,26,27].

The mechanical forms and action positions of nanostructures in injection molding are similar to those of nano-imprint technology. Yang et al. [28] studied the nano-imprint of polymethyl methacrylate (PMMA) using molecular dynamics simulation, and the results showed that the potential energies at the beginning and end of the imprinting were different to some extent; they were not completely released, resulting in residual stress. Kang et al. [29,30] pointed out that the aspect ratio and shape of the mold insert had different effects on the stress distribution of the structure. Fang et al. [31] believed that the higher the temperature, the greater the shear strain of the atoms around the mold.

In this study, molecular dynamics simulation was adopted to study the mechanism of residual stresses in micro-injection molding. The variation of dynamic stress, the morphological and structural evolution of molecular chains and the influence of mold inserts with different aspect ratios on the distribution of residual stresses in injection molding were investigated. The potential energy and radius of gyration were introduced to explore the flow properties of molecular chains, and the formation mechanism of residual stresses in injection molding was verified.

## 2. Materials and Methods

### 2.1. Model Constructing

**Figure 1:**
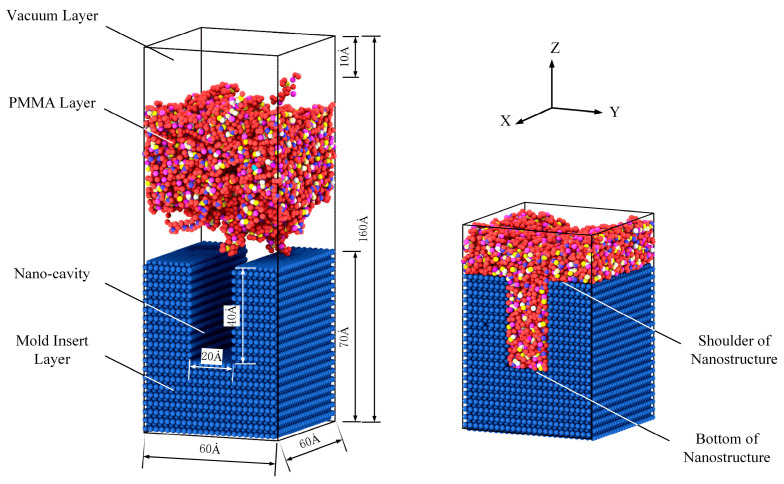
Initial amorphous model of simulation.

Considering its good filling performance and excellent light transmission, PMMA material was selected for the simulation. The initial amorphous model of simulation is shown in Figure 1, including a mold insert layer, a polymer layer and a vacuum layer, with a box of 60 × 60 × 160 Ȧ. The mold insert layer was composed of nickel (Ni) atoms, which had an FCC (face center cubic) structure with a (1 0 0) plane and a height of 70 Ȧ. A groove of 20 × 40 Ȧ was cut to form the nano-cavity, with an aspect ratio of 2.0. The PMMA layer was constructed in a configuration with a polymerization degree of 20 and chain number of 50. A periodic polymer cell with a temperature of 298 K and a density of 1.18 g/cm^3^ was established. Then, a cyclic annealing treatment with a high temperature of 500 K and a low temperature of 298 K was carried out under the NVT (the number of particles, the volume and the temperature of the system are kept constant) ensemble to form a polymer system in a molten state. A vacuum layer with a height of 10 Ȧ was applied over the PMMA layer to prevent the polymers from coming into contact with the air. In addition, the *x*, *y* and *z* axes were assigned to the periodic, periodic and free boundary conditions, respectively. Thus, the initial model of the simulation was established.

### 2.2. Force Field

A consistent valence force field (CVFF) was adopted to represent the intermolecular and nonbonding interactions between PMMA atoms. As shown in Equation (1), it was composed of bond stretching potential, angular bending potential, torsion potential and nonbonding interaction. Lennard–Jones 12–6 potentials were adopted to describe the nonbonding interaction between the atoms in the PMMA layer and the nickel atoms in the mold insert layer.

(1) $$\begin{array}{l} U_{total}=U_{bond}+U_{angle}+U_{torsion}+U_{nonbond}\\ =k_{b}{\left(l-l_{0}\right)}^{2}+k_{a}{(\theta -\theta_{0}}^{2}+k_{t}(1+\cos(n_{\phi }-\phi_{0}))+4\varepsilon_{ij}\left({\left(\frac{\sigma_{ij}}{r_{ij}}\right)}^{12}-{\left(\frac{\sigma_{ij}}{r_{ij}}\right)}^{6}\right) \end{array}$$

where $k_{b}$, $k_{a}$, and $k_{t}$ were the stiffness constants of bond stretching potential, angular bending potential and torsion potential. l, θ, $n_{\phi }$and $l_{0}$, $\theta_{0}$, $\phi_{0}$ were the bond length, bond angle, torsion angle and the average values of them, respectively. $\sigma_{ij}$ and $\varepsilon_{ij}$ were the two Lennard–Jones potential parameters that defined the nonbonding interaction, which represented the well depth and zero-potential distance of the Lennard–Jones potential. Finally, $r_{ij}$ was the distance between the atoms i and j. The cut-off distance of the nonbonding interaction was set to be 1.25 nm.

### 2.3. Simulation Procedure

**Figure 2:**
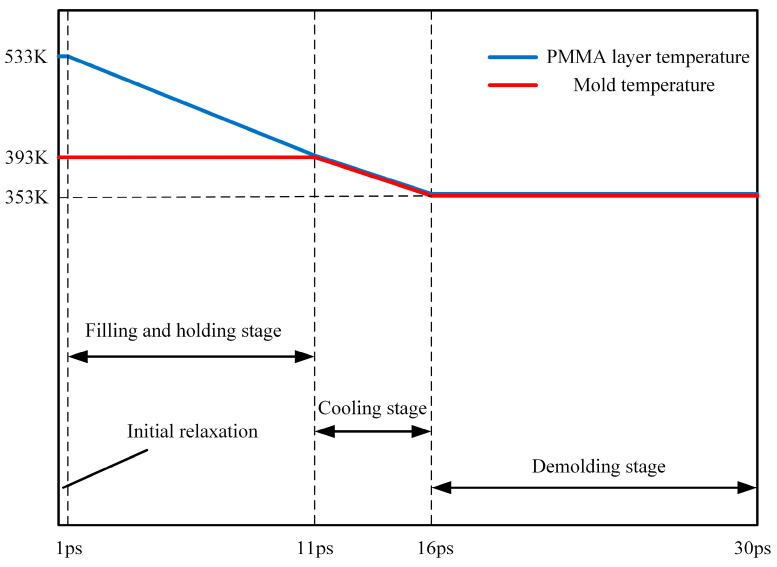
Time and temperature settings of simulation procedure for the injection molding process.

The simulations were performed using a large-scale atomic/molecular massively parallel simulator (LAMMPS), which was an open-source molecular dynamics package in a computer cluster. After 1.0 ps initial relaxation, the simulation procedure for the injection molding process included filling, packing, cooling and demolding, as shown in Figure 2. It was assumed that the Ni mold insert was rigid because Young’s modulus of the PMMA polymer was negligible relative to that of Ni, so all Ni atoms were fixed in their initial positions in the simulation. Prior to the simulation of injection molding, the PMMA layer was heated to 533 K, above the melting temperature, and relaxed for a period to achieve the optimal initial state. During the filling stage, each PMMA atom was applied with a force of 1.0 kcal/mol·Ȧ (equal to 0.07 nN) to ensure that the melt smoothly filled the nano-cavity. When a clear nanostructure profile was formed, the simulation procedure immediately turned into the packing stage, with the PMMA atoms becoming more and more compact under the packing pressure. Next, it entered the cooling stage, as the nanostructure cooled to 353 K with the mold insert. Under a demolding force of 1.0 kcal/mol Ȧ in the opposite direction, the PMMA nanostructure moved upward and finally out of the cavity. Thus, an entire injection molding process was completed. In the whole simulation process, the time step, the total step number and the ensemble were 0.2 fs, 150,000 and NVT, respectively.

## 3. Results and Discussion

### 3.1. Dynamic Stresses during Injection Molding

In order to investigate the formation mechanism of residual stresses during the injection molding process, it was necessary to observe and analyze the dynamic stresses in this process. By calculating the stress and volume of each atom, the stress contour was obtained. The stress was analyzed by the von Mises stress, which was a combination of normal and shear stress, and the equivalent stress was calculated by Equation (2).

(2) $$\sigma_{von}^{2}=3(\sigma_{xy}^{2}+\sigma_{yz}^{2}+\sigma_{xz}^{2})+\frac{1}{2}[{\left(\sigma_{xx}-\sigma_{yy}\right)}^{2}+{\left(\sigma_{yy}-\sigma_{zz}\right)}^{2}+{\left(\sigma_{xx}-\sigma_{zz}\right)}^{2}]$$

**Figure 3:**
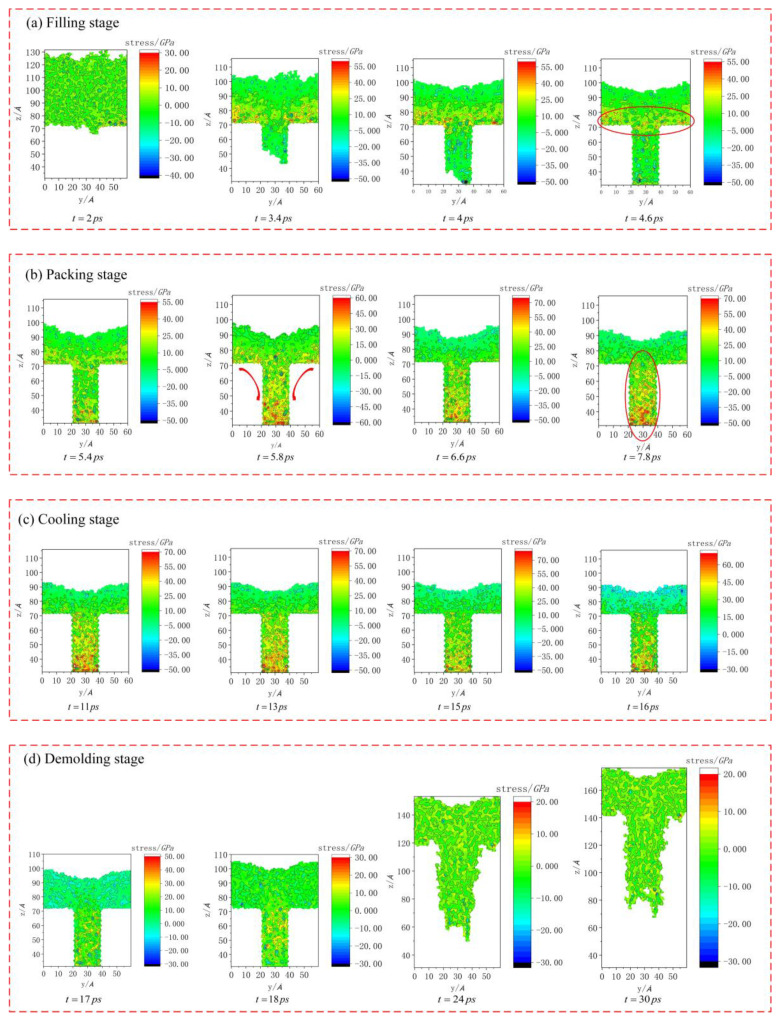
Snapshots of stress changes during the injection molding process.

As shown in Figure 3, there was a series of snapshots of stress changes during the injection molding process. The dynamic stress changes were mainly divided into four injection molding stages. However, the filling and packing stages could not be completely divided in the simulation, and these two stages could only be distinguished according to the PMMA layer completely entering the mold cavity to form a clear nanostructure profile.

It could be seen that the internal stress distribution of the polymer was nearly uniform, and the overall stresses after initial relaxation were relatively small. With the progress of the filling stage, the stresses of the nanostructure gradually increased, and the stress concentration appeared at the shoulders of the nanostructure at 4.6 ps, showing obvious surface compressive stresses. Then, the density of the PMMA layer increased continuously under the packing pressure, forming a complete nanostructure. With the progress of the packing stage, the internal stresses of the nanostructure further increased. At 5.8 ps, the stress concentration area began to transfer from the shoulders to the bottom. Then, during the cooling stage, the overall stresses of the nanostructure remained stable and fluctuated to a certain extent. Finally, under the demolding force, the nanostructure was gradually separated from the cavity. In the demolding stage, the overall stresses of the structure decreased sharply. When the demolding stage was carried out to 18 ps, the stress concentration area of the structure began to spread, and eventually the stress distribution was relatively uniform. At this point, it could be seen that there were considerable stresses in the nanostructure, resulting in the residual stresses and large deformation of the structure.

**Figure 4:**
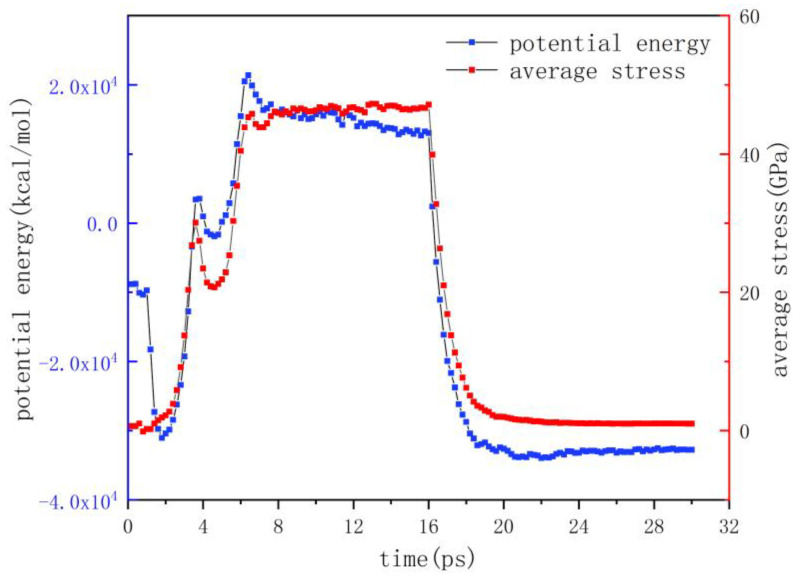
Variation of potential energy and average stress.

Potential energy is an important parameter reflecting the actual stress changes. The changes in the molecular structure in the flow process, such as the compression or stretching of the molecular chain, are signs of stress formation, which lead to further changes in the related potential energy, such as bond stretching potential, angular bending potential and torsion potential. At the same time, Figure 4 shows the change curves of the potential energy and average stress of the PMMA layer during injection molding. It could be seen that the changes in both were basically the same. Therefore, we had sufficient evidence to show that the potential energy was closely related to the formation of stresses.

By analyzing the changes in potential energy and average stress, we could roughly infer the changes of the molecular chains. The results showed that the potential energy decreased to zero at 1.8 ps and increased in the opposite direction under the injection pressure, and the average stress increased from around 0. Combined with the stress snapshots, it could be assumed that after relaxation, the stretched molecular chains were gradually compressed on the shoulders of the nanostructure, with the bond spacing decreasing as if a spring were compressed. When the PMMA layer continued to move down, the potential energy and average stress reached the first peak and decreased at 3.6 ps, which was caused by the molecular chains that were compressed on the shoulder beginning to align along the flow direction and being forced to stretch. With the further application of injection pressure, both of them began to increase rapidly, reaching the second peak at 6.4 ps, which was the maximum value in the injection molding process. At this point, the molecular chains at the bottom of the nanostructure started to be compressed and stored again, due to the limitation of the cavity.

Then, the potential energy of the molecular chain decreased slowly in the stages of packing and cooling, which may indicate that the temperature was the main factor controlling this process. With the decrease in the temperature, the oscillation frequency of the particles decreased, and the average stress remained within a certain range. Finally, the potential energy and the average stress decreased sharply due to the stretch and deformation of the molecular chains, under the combination of the release force, the adhesion force between the mold insert and the polymer and the elastic recovery force of the nanostructure. Eventually, the average stress was slightly higher than the initial stress, and the potential energy was not fully released. The residual stresses still existed in the structure.

### 3.2. Evolution of Molecular Morphology and Structure

The migration of molecular chains and the change in molecular orientation in the PMMA layer during injection molding were studied. The evolution law of molecular morphology and structure were discussed, which helped to reveal the formation mechanism of stresses in the molding process from the molecular level.

**Figure 5:**
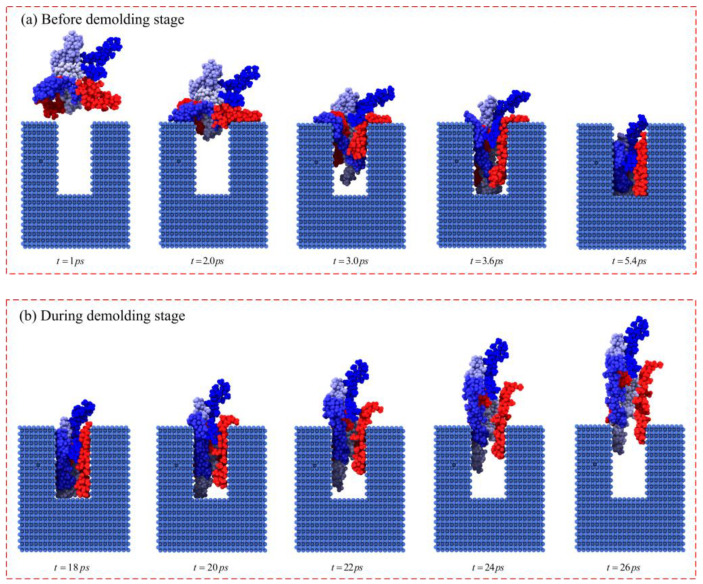
Morphology and structure evolution of molecular chains.

The morphology and structure evolution of the molecular chains were output by an open visualization tool (OVITO), as shown in Figure 5. The colors of some representative molecular chains were different, so it was easy to observe the changes in the molecular chains during the flow process. Before the injection molding, the stretching shape and the orientation of each molecular chain were disordered and isotropic. At 2.0 ps, the molecular chains that first reached the shoulders of the nanostructure were compressed, which was possibly the reason that the stress concentration area appeared first in the shoulders. Then, at 3.0 ps, the molecular chains that had compressed on the shoulders expanded and stretched, overcoming the steric hindrance, and oriented along the flow direction. The concentration area of the shoulders of the nanostructure gradually disappeared. Subsequently, the molecular chains were constantly pressed into the nano-cavity at 3.6 ps, and then those that reached the bottom of the nanostructure started to be compressed, with the stress concentration area transferred from the shoulders to the bottom. At this point, we could clearly observe that the morphology and structure of each polymer chain exhibited anisotropic characteristics and were oriented in the direction of flow. During the demolding stage, with the constant release of stresses, the molecular chains were continuously stretched, and there was an obvious displacement between the molecular chains at 24 ps, which was the reason for the deformation of the nanostructure.

**Figure 6:**
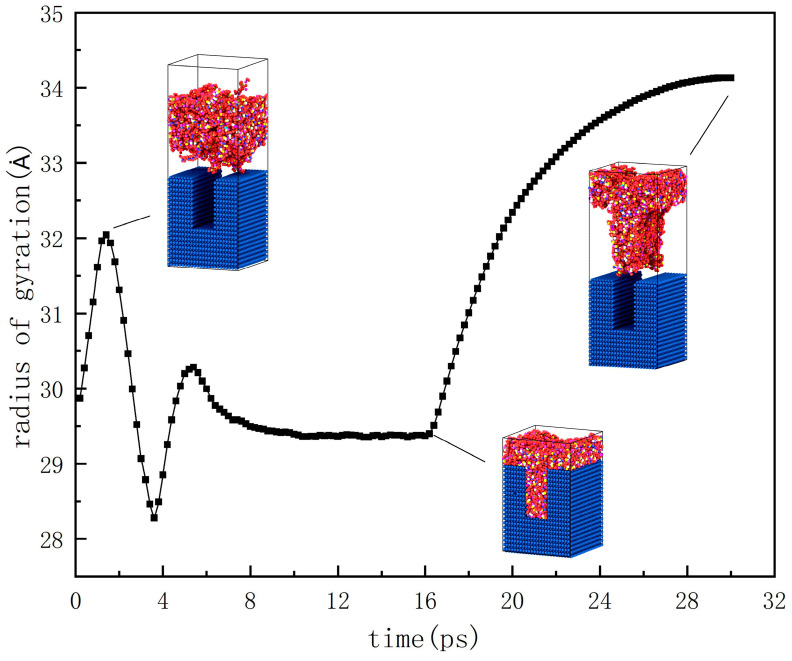
Variation of the radius of gyration with time.

The radius of gyration is a crucial parameter to characterize the spatial shape distribution of molecular chains, so it could be used to quantitatively characterize the morphology and structure of molecular chains. Figure 6 demonstrates the variation of the gyration radius with time. In the relaxation period, each molecular chain continuously stretched with the increase in temperature, and its radius of gyration increased, reaching a peak value of 32 Ȧ at 1.4 ps. With the molecular chains on the shoulder of the nanostructure compressed and entangled, the radius of gyration slowly decreased to 28.2 Ȧ at 3.6 ps. At this point, the energy inside the polymer kept accumulating, and the molecular chains with a higher internal energy began to orientate along the flow direction under the injection pressure and the limit of the cavity wall. Therefore, the radius of gyration increased again with the extension of the molecular chains. Moreover, it reached 30.3 Ȧ when filling to 5.4 ps.

Then, the molecular chains reaching the bottom were gradually compressed and frozen in the nanostructure, at which point the radius of gyration decreased and remained stable at around 29.3 Ȧ. This value was greater than the valley value of 28.2 Ȧ, which can be explained by the fact that the orientation behavior of the molecular chains was dominant, relative to the compression behavior. Most molecular chains were rapidly stretched and their radius of gyration correspondingly increased sharply.

Through the description of the evolution of the molecular morphology and structure, it can be seen that the essence of stress formation was the unbalanced conformation of molecular chains during the molding process, such as ordered orientation, compression and entanglement behavior. This unbalanced conformation could not be immediately restored to the equilibrium conformation suitable for environmental conditions, and it was frozen in the nanostructure, where it was stored as potential energy. However, this unbalanced conformation was also a kind of partially reversible deformation. Therefore, during the demolding stage, the compressed unstable conformation would be automatically converted into free stable conformation. At the same time, the stored potential energy would be transformed into the elongation and migration of the molecular chains. At this point, the whole injection molded structure would be deformed.

### 3.3. Effect of the Aspect Ratio of the Nano-Cavity

Nanostructures with different aspect ratios vary in their flow rate, flow resistance and other factors, so that the aspect ratio affects the formation and distribution of residual stresses. In order to further study the effect of aspect ratio on stress formation, the aspect ratio was controlled by adjusting the cavity depth in the simulation.

**Figure 7:**
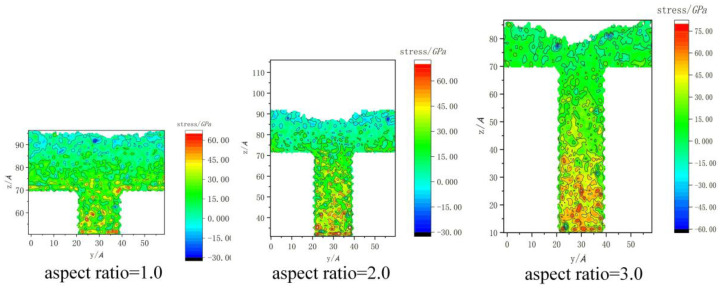
Stress distribution of nanostructures with different aspect ratios before demolding.

The stress distribution values of the nanostructures with different aspect ratios before demolding are compared in Figure 7. It can be seen that three structures with different aspect ratios were all filled in the full outlines. The results show that the overall stress of the structure increased with the increase in aspect ratio, and the maximum stress of the nanostructures with three aspect ratios were 68.3 GPa, 83 GPa and 86 GPa, respectively. Due to the increase in aspect ratio, more polymer and larger deformation were required to form a complete nanostructure. Therefore, greater injection and packing pressure were needed, resulting in an increase in the overall stress. In addition, when the aspect ratio was 1.0, the stress concentration areas were mainly distributed at the bottom and shoulders of the nanostructure. With the increase in the aspect ratio to 2.0, the stress concentration area was transferred to the bottom. When the aspect ratio was 3.0, the spread stress concentration area also appeared at the bottom, and the stress value was obviously larger than the other two.

**Figure 8:**
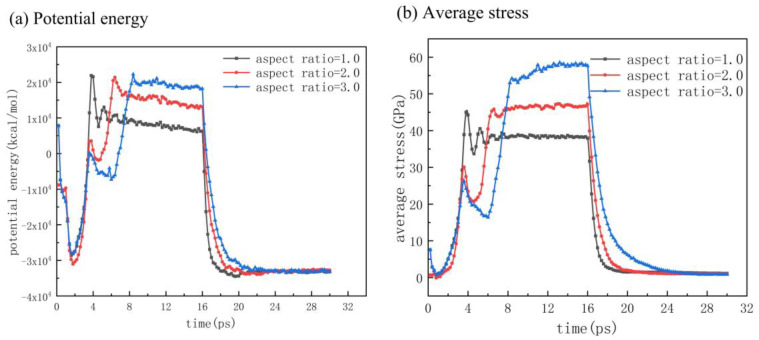
Variation of potential energy (**a**) and average stress (**b**) with different aspect ratios.

The change curves of potential energy and average stress are shown in Figure 8. As the aspect ratio increased, the time required to reach the packing stage and complete the demolding stage in injection molding also increased. Considering the increase in cavity depth, the PMMA layer needed a longer time to arrive at the bottom and eject from the cavity. With the increase in aspect ratio, the potential energy and average stress were increased during the packing stage, indicating that more molecular chains were in the compression state.

**Figure 9:**
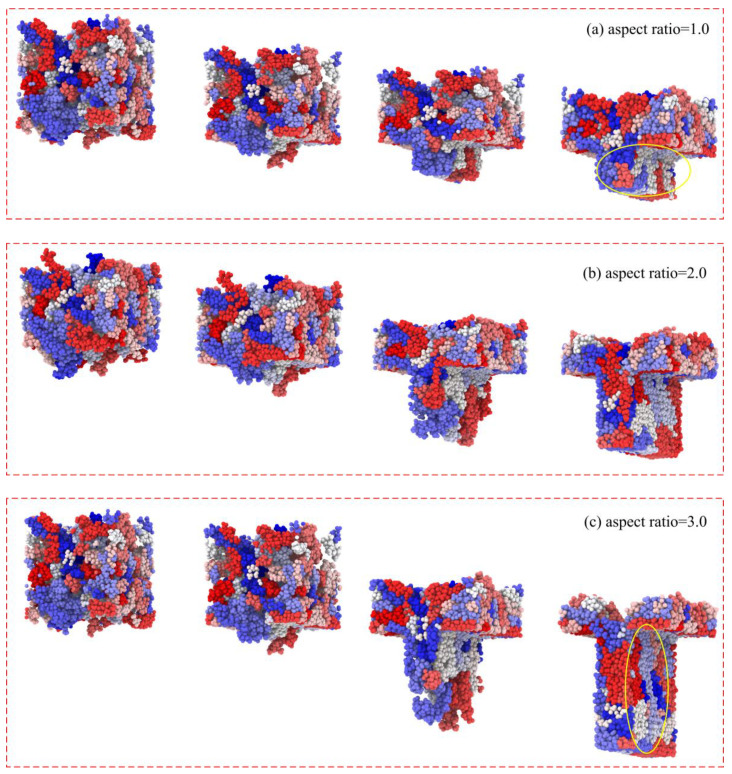
Morphology and structure of molecular chains with different aspect ratios.

To further investigate the influence of stress distribution in nanostructures with different aspect ratios, the morphology and structure evolution of molecular chains in the molding process are shown in Figure 9. When the aspect ratio was 1.0, some molecular chains that were compressed at the shoulders had reached the bottom before their orientation along the flow direction due to the limitation of cavity size. This may be the reason for the stress concentration area appearing at the shoulders and bottom of the nanostructure. At the same time, it could be seen that the orientation of the molecular chains along the flow direction was not clear, so the overall stress in the nanostructure was relatively small. When the aspect ratio reached 2.0 and 3.0, more molecular chains were oriented along the flow direction and were compressed and tangled at the bottom of the nanostructure, resulting in a larger stress concentration area. In this case, the anisotropy of the nanostructure was more obvious and the overall stress inside was greater.

**Figure 10:**
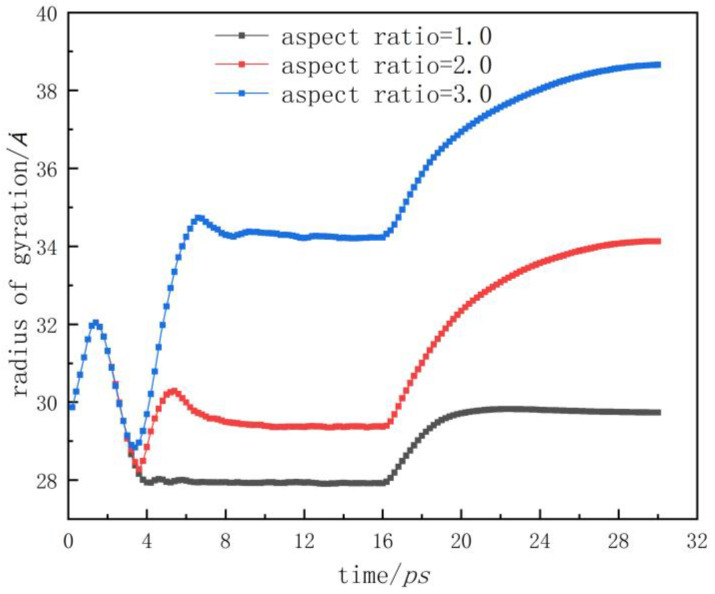
Radius of gyration with different aspect ratios.

The changes in the radii of gyration in nanostructures with different aspect ratios are shown in Figure 10. The radii of gyration in the filling stage were constantly decreased, and the three curves basically coincided. With aspect ratios of 2.0 and 3.0, the gyration radii of the nanostructures increased after 3.4 ps, but they still decreased when the aspect ratio was 1.0. This indicated that most molecular chains were in the compression state, and it also verified the above explanation about the evolution of the molecular chains. Then, the gyration radii of the nanostructures with aspect ratios of 2.0 and 3.0 began to decrease slowly at 5.4 ps and 6.6 ps, respectively. At this point, the expansion and stretching behaviors of the molecular chains were stronger than the compression and entanglement behaviors, indicating the longer time needed to reach the packing stage. Additionally, in the stages of packing and cooling, the radii of gyration were stable at around 27.9 Ȧ, 29.3 Ȧ and 34.2 Ȧ, respectively. This showed that with the increase in aspect ratio, more molecular chains were oriented along the flow direction, which led to the increase in the gyration radius. In the demolding stage, the radii of gyration increased sharply, and the increasing trend was proportional to the aspect ratio.

**Figure 11:**
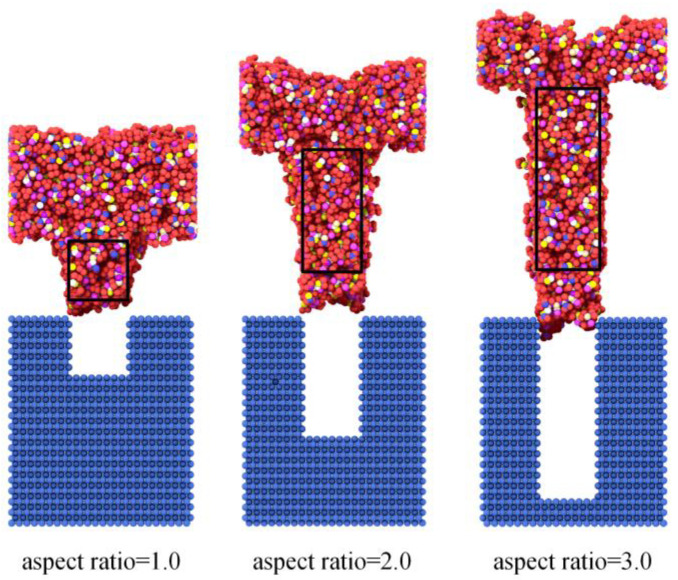
Deformations of nanostructures with different aspect ratios.

**Figure 12:**
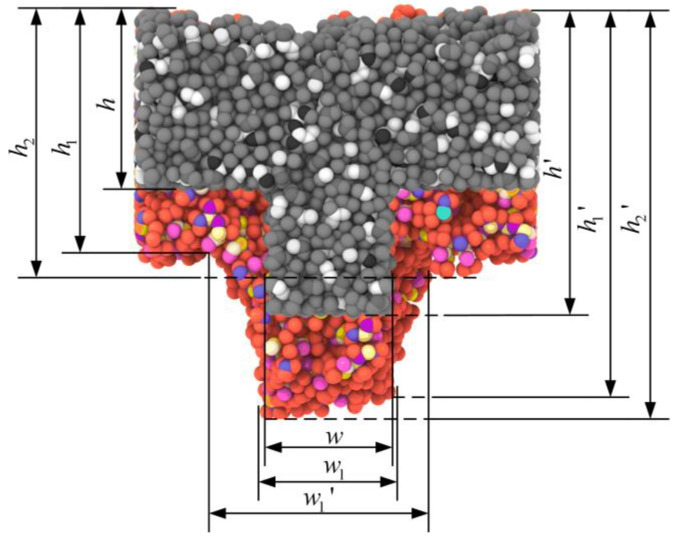
Calculation of elastic recovery percentage.

The most direct effect of the internal stresses was the quality of the nanostructures after demolding. There were more or less deformations of the nanostructures after demolding, as shown in Figure 11, where the black rectangle represents the shape of the nanostructure before demolding, and the whole nanostructure is widened and elongated in Figure 11. It could be observed that, with the increase in aspect ratio, the deformation of the nanostructures became more serious than that before demolding. Therefore, the degree of deformation after demolding could be obtained numerically by calculating the elastic recovery percentage (Equation (3)). The related symbols in Equation (3) are also demonstrated in Figure 12. The shapes of the nanostructure before and after demolding are represented by the dark and light parts in Figure 12, respectively.

(3) $$\eta =\left|\frac{h_{1}+h_{2}}{2h}\right|•\left|\frac{w_{1}+w_{2}}{2w}\right|•\left|\frac{{h_{1}}^{\prime }+{h_{2}}^{\prime }}{2h^{\prime }}\right|$$

where $h_{1}$, $h_{2}$, $w_{1}$, $w_{2}$, ${h_{1}}^{\prime }$ and ${h_{2}}^{\prime }$ represent the maximum and minimum values of the height and width of the bottom and the height of the shoulder of the nanostructure after demolding, respectively. h, wand $h^{\prime }$ represent the values of the height and width of the bottom and the height of the shoulder of the nanostructure before demolding.

With aspect ratios of 1.0, 2.0 and 3.0, the elastic recovery percentages were calculated as 288%, 297% and 311%, respectively. Therefore, as the aspect ratio increased, the stresses also increased, resulting in an increase in the elastic recovery after demolding and a larger deformation.

## 4. Conclusions

In this study, molecular dynamics simulation was used to investigate the formation mechanism of residual stresses in the injection molding of the PMMA polymer. The changes in stress, potential energy and average stress were analyzed in order to explore the dynamic stress during the molding process. The migration and orientation were discussed in order to characterize the evolution of molecular chains. It was found that the potential energy was related to the formation of stress, and its variation trend was basically consistent with the average stress. The essence of stress formation was unbalanced conformation, such as orientation, compression and entanglement behaviors, and the orientation of molecular chains changed from anisotropy to isotropy. By comparing the stress distribution of nanostructures with the different aspect ratios of 1.0, 2.0 and 3.0, the overall stress and degree of deformation increased with an increase in aspect ratio. In addition, the elastic recovery percentages were calculated as 288%, 297% and 311%, respectively. With the increase in aspect ratio, a larger elastic recovery percentage indicated a larger deformation caused by the residual stresses. Consequently, the stress relaxation behaviors after the molding process will be investigated in our future work.

## Nomenclature

$k_{b}$	Stiffness constant of bond stretching potential
$k_{a}$	Stiffness constant of angular bending potential
$k_{t}$	Stiffness constant of torsion potential
l, $l_{0}$	Bond length and equilibrium bond length
θ, $\theta_{0}$	Bond angle and equilibrium bond angle
$n_{\phi }$, $\phi_{0}$	Torsion angle and equilibrium torsion angle
$\sigma_{ij}$	Zero-potential distance of Lennard-Jones
$\varepsilon_{ij}$	Well depth of Lennard-Jones
$r_{ij}$	Distance between the atom i and j
$h_{1}$, $h_{2}$	Maximum and minimum values of the height of bottom after demolding
$w_{1}$, $w_{2}$	Maximum and minimum values of the width of bottom after demolding
${h_{1}}^{\prime }$, ${h_{2}}^{\prime }$	Maximum and minimum values of the height of shoulder after demolding
h, w	Values of the height and width of bottom before demolding
$h^{\prime }$	Values of the height of shoulder before demolding

## References

[1] Wu J., He Z., Chen Q., Lin J. (2016). Biochemical analysis on microfluidic chips. TrAC Trends Anal. Chem..

[2] Kamaly N., Fredman G., Fojas J.J.R., Subramanian M., Choi W.I., Zepeda K., Vilos C., Yu M., Gadde S., Wu J. (2016). Targeted interleukin-10 nanotherapeutics developed with a microfluidic chip enhance resolution of inflammation in advanced atherosclerosis. ACS Nano.

[3] Weng C., Wang F., Zhou M., Yang D., Jiang B. (2018). Fabrication of hierarchical polymer surfaces with superhydrophobicity by injection molding from nature and function-oriented design. Appl. Surf. Sci..

[4] Gupta M.C., Ungaro C., Foley J.J., Gray S.K. (2018). Optical nanostructures design, fabrication, and applications for solar/thermal energy conversion. Sol. Energy.

[5] Huang H.X., Wang X. (2019). Biomimetic fabrication of micro-/nanostructure on polypropylene surfaces with high dynamic superhydrophobic stability. Mater. Today Commun..

[6] Stormonth-Darling J.M., Saeed A., Reynolds P.M., Gadegaard N. (2016). Injection molding micro- and nanostructures in thermoplastic elastomers. Macromol. Mater. Eng..

[7] Kim B., Min J. (2017). Residual stress distributions and their influence on post-manufacturing deformation of injection-molded plastic parts. J. Mater. Process. Technol..

[8] Macías C., Meza O., Pérez E. (2015). Relaxation of residual stresses in plastic cover lenses with applications in the injection molding process. Eng. Fail. Anal..

[9] Jeong S., Kim J.M., Baig C. (2017). Effect of chain orientation and stretch on the stress overshoot of entangled polymeric materials under start-up shear. Macromolecules.

[10] Jarecki L., Misztal-faraj B. (2017). Non-linear stress-orientation behavior of flexible chain polymers under fast elongational flow. Eur. Polym. J..

[11] Salmoria G.V., Vieira L.F., Gindri I.M., Roesler C.R.M., Fancello E.A. (2018). Properties of injection-molded poly (l-co-d,l-lactic acid) using different melt temperatures and stress concentrator in the specimen geometry. Int. J. Adv. Manuf. Technol..

[12] Yue P. (2019). Molecular orientation distribution in PC products analyzed by birefringence. Phys. B Phys. Condens. Matter.

[13] Weng C., Lee W.B., To S. (2009). Birefringence techniques for the characterization of residual stresses in injection-moulded micro-lens arrays. Polym. Test..

[14] Ghiam F., White J.L. (1991). Phase morphology of injection-molded blends of nylon-6 and polyethylene and comparison with compression molding. Polym. Eng. Sci..

[15] Lin C.-M., Hsieh H.-K. (2017). Processing optimization of Fresnel lenses manufacturing in the injection molding considering birefringence effect. Microsyst. Technol..

[16] Liparoti S., Sorrentino A., Titomanlio G. (2015). Rapid control of mold temperature during injection molding process: Effect of packing pressure. AIP Conf. Proc..

[17] Kim C.H., Youn J.R. (2007). Determination of residual stresses in injection-moulded flat plate: Simulation and experiments. Polym. Test..

[18] Polymeris G.S., Vlachos N., Khan A.U., Hatzikraniotis E., Lioutas C.B., Delimitis A., Pavlidou E., Paraskevopoulos K., Kyratsi T., Kyratsi T. (2015). Nanostructure and doping stimulated phase separation in high-ZT Mg2Si0.55Sn0.4Ge0.05 compounds. Acta Mater..

[19] Wang S., Javadpour F., Feng Q. (2016). Molecular dynamics simulations of oil transport through inorganic nanopores in shale. Fuel.

[20] Zhou M., Jiang B., Weng C. (2016). Molecular dynamics study on polymer filling into nano-cavity by injection molding. Comput. Mater. Sci..

[21] Yeh I.-C., Andzelm J.W., Rutledge G.C. (2015). Mechanical and structural characterization of semicrystalline polyethylene under tensile deformation by molecular dynamics simulations. Macromolecules.

[22] Wang B.-B., Xu Z.-M., Wang X.-D., Yan W.-M. (2018). Molecular dynamics investigation on enhancement of heat transfer between electrified solid surface and liquid water. Int. J. Heat Mass Transf..

[23] Thomas T.M., Vinod N. (2019). Convective heat transfer between liquid argon flows and heated carbon nanotube arrays using molecular dynamics. J. Appl. Fluid Mech..

[24] Zhang J., Hong Y., Liu M., Yue Y., Xiong Q., Lorenzini G. (2017). Molecular dynamics simulation of the interfacial thermal resistance between phosphorene and silicon substrate. Int. J. Heat Mass Transf..

[25] Xu G., Wang H. (2016). Molecular dynamics study of interfacial mechanical behavior between asphalt binder and mineral aggregate. Constr. Build. Mater..

[26] Xu G., Wang H. (2016). Study of cohesion and adhesion properties of asphalt concrete with molecular dynamics simulation. Comput. Mater. Sci..

[27] Min K., Kim Y., Goyal S., Lee S.H., McKenzie M., Park H., Savoy E.S., Rammohan A.R., Mauro J.C., Kim H. (2016). Interfacial adhesion behavior of polyimides on silica glass: A molecular dynamics study. Polym..

[28] Yang S., Yu S., Cho M. (2014). Influence of mold and substrate material combinations on nanoimprint lithography process: MD simulation approach. Appl. Surf. Sci..

[29] Kang J.-H., Kim K.-S., Kim K.-W. (2007). Molecular dynamics study of pattern transfer in nanoimprint lithography. Tribol. Lett..

[30] Kang J.-H., Kim K.-S., Kim K.-W. (2010). Molecular dynamics study on the effects of stamp shape, adhesive energy, and temperature on the nanoimprint lithography process. Appl. Surf. Sci..

[31] Fang T., Wu C., Chang W., Chi S. (2009). Effect of thermal annealing on nanoimprinted Cu—Ni alloys using molecular dynamics simulation. Appl. Surf. Sci..

